# Occurrence of *Cis*-11,12-Methylene-Hexadecanoic Acid in the Red Alga *Solieria pacifica* (Yamada) Yoshida

**DOI:** 10.3390/molecules26082286

**Published:** 2021-04-15

**Authors:** Gwang-Woo Kim, Jae-Man Sim, Yutaka Itabashi, Min-Jeong Jung, Joon-Young Jun

**Affiliations:** 1 Division of Marine Bio Convergence, Gangneung Science and Industry Promotion Agency, Gangneung 25451, Korea; no-1duke@hanmail.net (G.-W.K.); sjman7514@hanmail.net (J.-M.S.); 2 Faculty of Fisheries Sciences, Hokkaido University, Hakodate 041-8611, Japan; 3 Japan Association for Inspection and Investigation of Foods including Fats and Oils, Tokyo 103-0007, Japan; 4 Division of Strategic Food Technology Research, Korea Food Research Institute, Gangneung 25440, Korea; ooojmj@nate.com

**Keywords:** red alga, solieriaceae, *Solieria pacifica*, cyclic fatty acid, *cis*-11,12-methylene-hexadecanoic acid, *cis*-11-hexadecenoic acid

## Abstract

Fatty acids in marine algae have attracted the attention of natural chemists because of their biological activity. The fatty acid compositions of the Solieriaceae families (Rhodophyceae, Gaigartinales) provide interesting information that unusual cyclic fatty acids have been occasionally found. A survey was conducted to profile the characteristic fatty acid composition of the red alga *Solieria pacifica* (Yamada) Yoshida using gas chromatography-mass spectrometry (GC-MS), infrared spectroscopy (IR), and proton nuclear magnetic resonance spectroscopy (^1^H-NMR). In *S. pacifica,* two cyclopentyl fatty acids, 11-cyclopentylundecanoic acid (7.0%), and 13-cyclopentyltridecanoic acid (4.9%), and a cyclopropane fatty acid, *cis*-11,12-methylene-hexadecanoic acid (7.9%) contributed significantly to the overall fatty acid profile. In particular, this cyclopropane fatty acid has been primarily found in bacteria, rumen microorganisms or foods of animal origin, and has not previously been found in any other algae. In addition, this alga contains a significant amount of the monoenoic acid *cis*-11-hexadecenoic acid (9.0%). Therefore, *cis*-11,12-methylene-hexadecanoic acid in *S. pacifica* was likely produced by methylene addition to *cis*-11-hexadecenoic acid.

## 1. Introduction

Marine macroalgae of the family Solieriaceae (Gigartinales, Rhodophyta) are frequently consumed as edible seaweeds and are known to produce carrageenan polysaccharides (mainly the sulfated galactoside *i*-carrageenan), which are used as a common ingredient in many dairy foods products, such as yogurt or chocolate milk [1,2]. Marine algae are known to contain long-chain polyunsaturated fatty acids (PUFA) such as omega-3 fatty acids and a valuable potential source of essential fatty acids, which are important for human and animal nutrition [3,4]. Red algae are especially rich in omega-3 essential fatty acids such as eicosapentaenoic acid (EPA) compared to brown and green algae [5]. 

Cyclic fatty acids are an unusual class of minor fatty acids that are generally produced by bacteria and less frequently by plants. Cyclopropane fatty acids, such as dihydrosterculic (9,10-methylene-octadecanoic acid) and lactobacillic (11,12-methylene-octadecanoic acid) acid, and ω-cyclohexyl fatty acids, such as 11-cyclohexylundecanoic and 13-cyclohexyltridecanoic acid, have been identified as minor components in the lipid profile of lactic acid bacteria and rumen microorganisms, respectively, and have been recently detected in foods of animal origin [6,7,8,9].

In seaweeds, a previous study [10] suggested that cyclic fatty acids from Solieriaceae families are of special interest. The fatty acid compositions of five Solieriaceae families (*Agardhiella tenera*, *Anatheca montagnei*, *Eucheuma cottonii*, *Eucheuma spinosum*, and *Meristotheca senegalensis*), are notably different from those of other red algae, indicating that the occurrence of the cyclopentyl fatty acids, 11-cyclopentylundecanoic acid (cy16:0) and 13-cyclopentyltridecanoic acid (cy18:0), and n-5 monounsaturated fatty acids (16:1n-5 and 18:1n-5), are characteristic metabolites. The content of cyclopentyl fatty acids as a measure of total fatty acids in these five Solieriaceae families is a relatively high at 6.5–6.8%. 

In addition to these characteristic fatty acids, in this study we identified another cyclopropane fatty acid, *cis*-11,12-methylene-hexadecanoic acid (cy17:0), from the Solieriaceae family member *Solieria pacifica* (Yamada) Yoshida (Japanese name, Mirin). This cyclopropane fatty acid has not previously been detected in any algal species. In the present study, we focused on identifying and characterizing *cis*-11,12-methylene-hexadecanoic acid in *S. pacifica.* We also identified two previously identified cyclopentyl fatty acids by generating picolinyl ester and 4,4-dimethyloxazoline (DMOX) derivatives.

## 2. Results and Discussion

### 2.1. GC Elution Profiles

**Figure 1:**
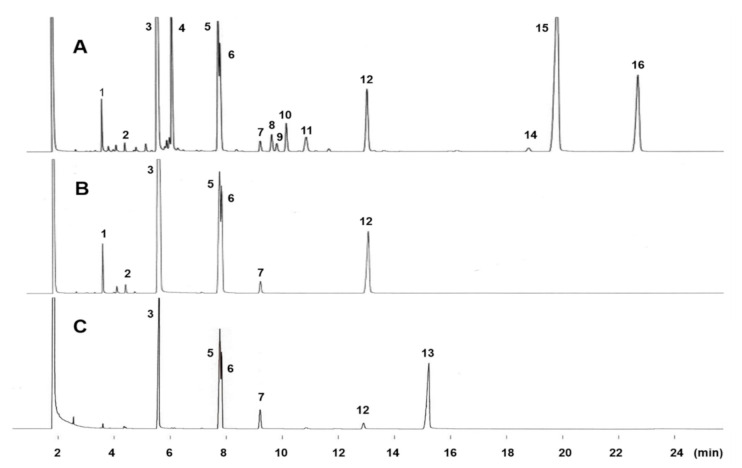
GC profiles of total fatty acid methyl esters from *Solieria pacifica* (**A**), their saturated fraction obtained by Ag^+^-TLC (**B**), and the hydrogenated FAME of total fatty acids (**C**) on Omegawax 320. The column temperature was programmed from 160 °C to 230 °C at 1 °C /min. Other GC conditions are given in text. Peak identification: **1** = 14:0; **2** = 15:0; **3** = 16:0; **4** = 16:1n-5; **5** = *cis*-11,12-methylene-hexadecanoic acid; **6** = 11-cyclopentylundecanoic acid; **7** = 18:0; **8** = 18:1n-9; **9** = 18:1n-7; **10** = 18:1n-5; **11** = 18:2n-6; **12** = 13-cyclopentyltridecanoic acid; **13 = 20:0; 14** = 20:3n-6; **15** = 20:4n-6; **16** = 20:5n-3.

The GC profiles of fatty acid methyl esters (FAME) from *S. pacifica* eluted from an Omegawax 320 column are shown in Figure 1. In addition to the typical saturated and unsaturated fatty acid peaks, unusual peaks marked 4, 5, 6, and 12 were observed in the chromatogram of FAME from the total lipids (Figure 1A). Complete separation of peaks 5 and 6 were achieved with a longer elution time when the column temperature was maintained at 160 °C (Supplementary Figure S1). Peaks 5, 6, and 12 appeared in the saturated FAME fraction obtained by Ag^+^-TLC (Figure 1B) and in the hydrogenated FAME of total fatty acids (Figure 1C). These results suggested that peaks 5, 6, and 12 were all saturated fatty acids. Peak 4 appeared in the monoenoic FAME fraction obtained by Ag^+^-TLC (chromatogram not shown). The equivalent chain length (ECL) values of peaks 4, 6, and 12 were 16.40, 17.44, and 19.45, respectively (Table 1), which are in good agreement with those of 11-hexadecenoic acid (16:1n-5), 11-cyclopentylundecanoic acid, and 13-cyclopentyltridecanoic acid, respectively, as obtained on Carbowax 20M, which is chemically similar to Omegawax and is likely to have similar chromatographic properties [10].

### 2.2. Structural Elucidation of cis-11,12-Methylene-Hexadecanoic Acid (Peak 5) 

**Figure 2:**
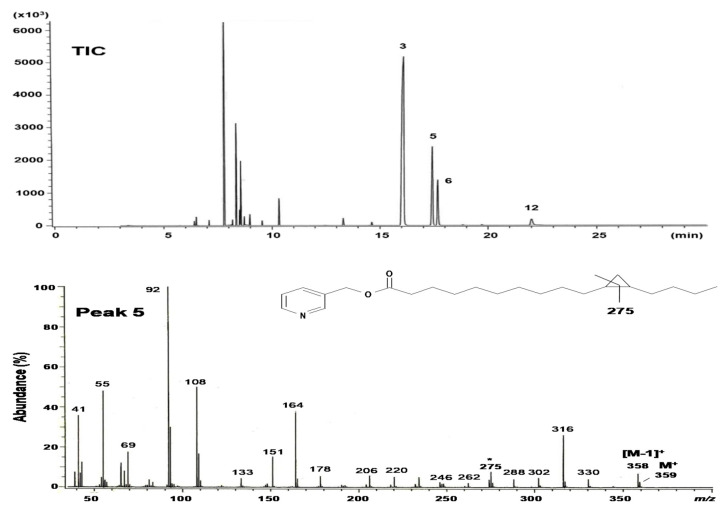

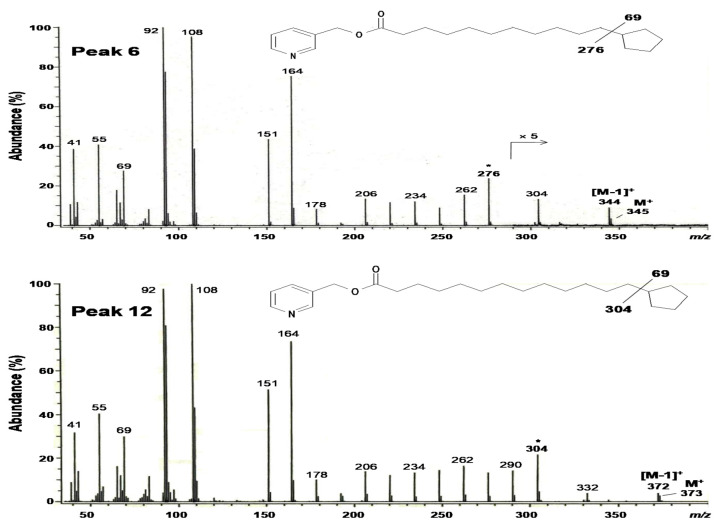
GC-MS profiles of the picolinyl ester derivatives of the saturated fraction of total fatty acids from *Solieria pacifica*. TIC, total ion chromatogram. Peak 3 = 16:0, Peak 5 = *cis*-11,12-methylene-hexadecanoic acid, Peak 6 = 11-cyclopentylundecanoic acid, and Peak 12 = 13-cyclopentyltridecanoic acid.

Figure 2 shows the gas chromatography-mass spectrometry (GC-MS) profiles of the picolinyl ester derivatives of the saturated fraction containing peaks 5, 6, and 12, which were obtained by Ag^+^-TLC from the total FAME of *S. pacifica* (Figure 1B). Similar to the separation of methyl esters on the Omegawax 320 column, picolinyl esters on an HP-5 column used for GC-MS gave a complete separation for peaks 5 and 6 under the conditions employed (Figure 2, TIC). The mass spectrum of peak 5 showed a molecular ion at *m/z* 359, suggesting a C16 cyclopropane acid, and prominent ions at *m/z* 92, 108, 151 (the McLafferty ion), and 164 derived from the picolinyl ester moiety. Picolinyl esters are the most useful derivatives for the characterization of cyclopropane and cyclopentyl fatty acids. In particular, the derivatives of cyclopropane fatty acids give distinctive cleavages that permit facile location of the cyclopropane ring [11,12]. In addition, though fatty acids possessing a cyclopropane ring have the same molecular mass as monoenes with the same carbon atoms, the mass spectra of their picolinyl esters are distinctive as they contain a prominent diagnostic ion with a characteristic mass formed by cleavage of the cyclopropane ring [13,14,15]. In the mass spectrum of peak 5 (Figure 2), this mass appeared at *m/z* 275, which represents a unique fragment containing C_11_ in the ring. Characteristic fragments of peak 5 were also found at *m/z* 262 and 302 from either side of the ring, and the abundance of the *m/z* 316 ion 41 amu higher than the *m/z* 275 ion. The abundant [M−1]^+^ ion at *m/z* 358 also served to indicate the presence of a cyclopropane acid. Taken together, peak 5 from this sample is likely a hexadecanoic acid with a cyclopropane ring between C_11_ and C_12_. 

**Figure 3:**
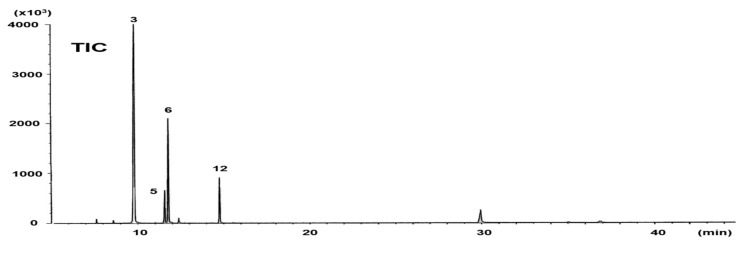

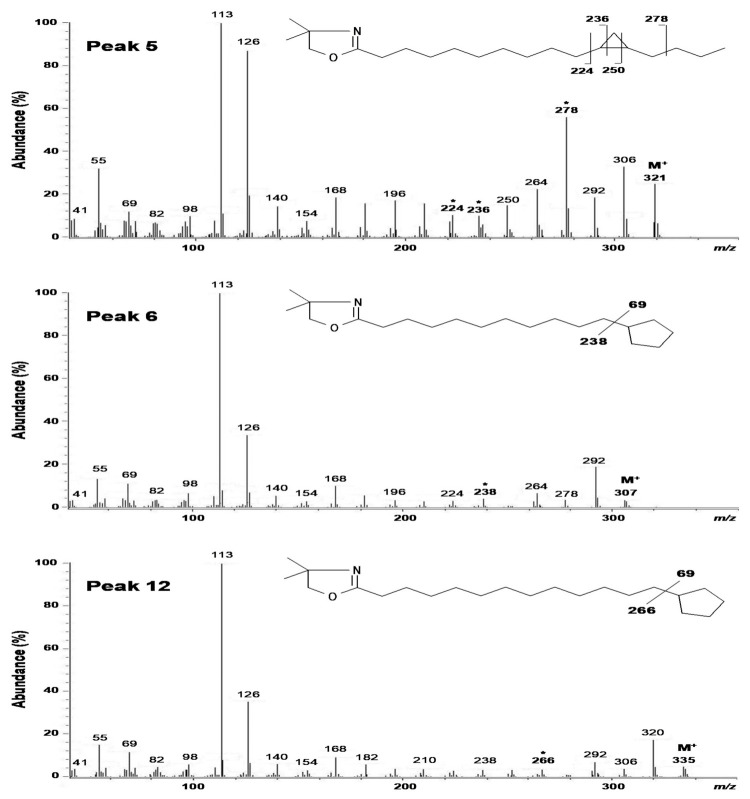
GC-MS profiles of the DMOX derivatives of the saturated fraction of total fatty acids from *Solieria pacifica*. TIC, total ion current chromatogram. For peak identification, see Figure 2.

The GC-MS profiles of the DMOX derivatives of the saturated fraction are shown in Figure 3. Clear separation for peaks 5 and 6 were obtained on the DB-23 column (Figure 3, TIC). The mass spectra of DMOX derivatives of fatty acids with cyclopropane rings are much less distinctive than those of picolinyl esters [12,15]. This is also the case for peak 5, but the assignment could be successfully completed despite this limitation. The spectrum of peak 5 yielded a molecular ion at *m/z* 321. Prominent ions at *m/z* 113 and 126 were observed, and the former (the McLafferty ion) represents cleavage between C_2_ and C_3_. Fragmentation patterns of cyclopropane fatty acids are comparable to those of monoenoic fatty acids, with differences only in the relative intensities of some ions [16,17]. A difference of 12 amu between *m/z* 224 and 236 indicates the position of the cyclopropane ring between C_11_ and C_12_ (Figure 3, Peak 5). This is accompanied by an intense ion fragment at *m/z* 278 derived from the *β*-cleavage ion, which unambiguously distinguishes between monoenoic and cyclopropane fatty acids [16,17,18]. 

**Figure 4:**
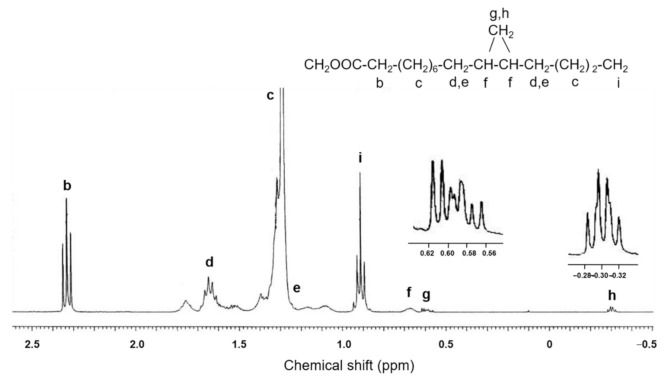
^1^H-NMR spectrum of the saturated fraction from total fatty acids of *Solieria pacifica* in the region −0.5 to 2.5 ppm. The peaks around 0.60 ppm and −0.30 ppm have been expanded.

Figure 4 shows the ^1^H-NMR spectrum of the saturated FAME fraction in the region of −0.5 to 2.5 ppm. The peaks at −0.30 and 0.60 ppm are consistent with a *cis* configuration of the two alkyl substituents on the cyclopropane ring, because *trans* cyclopropane com-pounds lack peaks in the region between −0.3 to −0.35 ppm [19].

The MS and NMR results together clearly establish that peak 5 is *cis*-11,12-methylene-hexadecanoic acid (cy17:0).

### 2.3. Structural Elucidation of 11-Cyclopentylundecanoic Acid (Peak 6), 13-Cyclopentyltridecanoic Acid (Peak 12), and cis-11-Hexadecenoic Acid (Peak 4)

The mass spectra of the picolinyl esters of peaks 6 and 12 show molecular ions at *m/z* 345 and 373, respectively (Figure 2). Fatty acids with a cyclopentyl ring have the same molecular mass as monoenes with the same carbon atoms, but their fragmentation pat-terns are different. The mass spectra of the picolinyl esters of saturated fatty acids with a terminal cyclopentyl group have gaps between the molecular ion and an ion at [M−69]^+^ due to ring loss [12]. This significant complementary ion appeared clearly in peaks 6 and 12, at *m/z* 276 and 304, respectively. The mass spectra of the DMOX derivatives for peaks 6 and 12 include molecular ions at *m/z* 307 and 335, respectively (Figure 3). As with the picolinyl esters, the key diagnostic ions for the cyclopentyl fatty acids were observed at *m/z* 238 (peak 6) and 266 (peak 12), which represent the loss of the terminal cyclopentyl ring as a radical fragment of 69 amu [17]. The mass spectra of the picolinyl ester and DMOX derivatives from peaks 6 and 12 were essentially the same as those reported previously [20,21]. These results demonstrate that peaks 6 is 11-cyclopentylundecanoic acid (cy16:0) and peak 12 is 13-cyclopentyltridecanoic acid (cy18:0).

The GC-MS of the picolinyl ester derivative of peak 4 (Figure 1A), which was prepared from the monoenoic FAME fraction obtained by Ag^+^-TLC, gave the molecular ion at *m/z* 345, suggesting a hexadecenoic acid (Supplementary Figure S2). The double bond position was determined by the distinctive fragmentation of the picolinyl ester. The key prominent ions with mass intervals of 26 amu were observed at *m/z* 262 and 288, which were formed by cleavage between C_11_ and C_12_ [17]. The Fourier transform infrared (FT-IR) spectrum of the methyl esters from the monoenoic acid fraction lacked an absorption band between 960 and 970 cm^−1^ (*trans* C-H out of plane deformation) (Supplementary Figure S3). This indicates that the double bonds in all monoenoic fatty acids in *S. pacifica* are of a *cis* configuration exclusively. These MS and IR results demonstrate that the structure of peak 4 is *cis*-11-hexadecenoic acid (16:1n-5).

### 2.4. Fatty Acid Composition

The fatty acid composition of the total lipids from *S. pacifica* is shown in Table 1. The predominant fatty acids were 16:0 (28.4% of total fatty acids) and 20:4n-6 (23.7%), which are typical fatty acid components in red algal lipids [22]. The content of cy17:0, cy16:0, cy18:0, 16:1n-5, and 18:1n-5 acids were 7.9, 7.0, 4.9, 9.0, and 1.6% of the total fatty acids, respectively. Although chemical characteristic of marine algae could be varied on their collecting season, region, or stage of development, even in same species, the fatty acid composition of *S. pacifica* is very different from other marine algae, as demonstrated by the presence of three cyclic fatty acids and 16:1n-5 acid.

Relatively high amounts of these uncommon fatty acids in the lipidome are of special interest. A previous study [10] reported the presence of significant amounts of 11-cyclopentylundecanoic (5.2–16.4% of total fatty acids), 13-cyclopentyltridecanoic (0.1–0.4%), 16:1n-5 (9.5–9.6%), and 18:1n-5 (1.6–4.6%) acids in five Solieriaceae families. However, to our knowledge, the significant amount of *cis*-11,12-methylene-hexadecanoic acid in *S. pacifica* has not been found in any other seaweed to date, although a very small amount of this cyclopropane acid (less than 0.1% of total fatty acids) has been tentatively identified in the lipids of killifish (*Fundulus heteroclitus*) [23], and found in the core of anaerobic marine sediment covered with a white mat of giant sulfide-oxidizing bacteria (*Beggiatoa* sp.) [24].

It is known that significant amounts of other cyclopropane acids, such as *cis*-9,10-methylene-octadecanoic acid, have been found in bacteria [25], trypanosomatid flagellates [26], millipedes [27], and higher plants [28,29]. A cyclic fatty acid such as *cis*-11,12-methylene-octadecanoic acid is known to be formed in small amount by intramolecular cyclization of unsaturated fatty acids during the refining and frying of vegetables [30] and animals [31]. Another uncommon monoenoic acid, 18:1n-5 (peak 10), a C2 elongation product of 16:1n-5, was also found in much lower amounts in *S. pacifica* (Table 1); however, *cis*-13,14-methylene-octadecanoic acid was not detected under the GC conditions employed. In our study, only one cyclopropane acid, *cis*-11,12-methylene-hexadecanoic acid, was found in the alga.

In a previous study, cyclopropane fatty acids, such as *cis*-9,10-methylene-hexadecenoic acid, *cis*-11,12-methylene-octadecanoic acid, and *cis*-9,10-methylene octadecanoic acid, have been found in membrane lipid of some bacteria [25]. Since the methylene group in cyclopropane acids originates from the methyl group of methionine in *S*-adenosyl methionine [24,25,28], the occurrence of *cis*-11,12-methylene-hexadecanoic acid in *S. pacifica* is likely to be associated with the significant amount of 16:1n-5 acid. The biological significance and occurrence of the cyclic fatty acids in *S. pacifica* remains to be elucidated through further study.

## 3. Materials and Methods

### 3.1. Samples, Lipid Extraction, and Analysis of Fatty Acids 

Fresh *S. pacifica* used in for this study was collected in August 2010 at Yuge Island, Ehime, Japan. The algal sample was well washed with filtered seawater and then heated for approximately 10 min in boiling water to deactivate hydrolytic enzymes, which decompose glycoglycerolipids and phospholipids to release free fatty acids (FFA) [32]. Approximately 100 g of the alga was finely cut (3–5 mm), and total lipids were extracted according to the method of Bligh and Dyer [33]. Fatty acids were converted to methyl esters (FAME) by heating at 95 °C for 1 h in 5% HCl/MeOH. FAME were purified on a small silica gel column using hexane-ether (9:1, *v*/*v*) as the mobile phase. The resulting FAME were confirmed on a silica gel 60 F_254_ aluminum TLC sheet (Merck, Darmstadt, Germany) using hexane-ether-acetic acid (70:30:1, *v*/*v*) as the developing solvent [34]. 

GC analysis of FAME was performed on a GC-14A gas chromatograph (Shimadzu, Kyoto, Japan) equipped with a flame ionization detector (FID) and an Omegawax 320 column (30 m × 0.32 mm i.d., 0.25 µm film thickness, Supelco, Bellefonte, PA, USA). Helium was used as the carrier gas at a flow rate of 1 mL/min, and the split ratio was 1:50. The injector and FID temperatures were 230 and 240 °C, respectively. The column temperature was programmed to increase from 160 to 230 °C at 1 °C/min. Peaks were monitored on a Chromatopac C-R6A integrator (Shimadzu) and identified using authentic standards (GLC-462, Nu-Chek Prep, Elysian, MN, USA), 11-hexadecenoic acid (92% *cis*, 8% *trans*, Matreya, PA, USA), equivalent chain length (ECL) values [34], and GC-MS, as described below.

### 3.2. Silver Ion-TLC (Ag^+^-TLC)

FAME were fractionated according to the degree of unsaturation by TLC on silica gel 60 F_254_-coated glass plates (Merck) immersed in 20% silver nitrate in acetonitrile for 15 min in the dark and activated at 100 °C for 1 h, using a mixture of hexane-ether-acetic acid (80:20:1, *v*/*v*) as the developing solvent. After development, the plate was sprayed with 0.2% 2,7-dichlorofluorescein in ethanol and viewed under UV light. The bands corresponding to saturates (R_f_ = 0.74) and monoenes (R_f_ = 0.63) were separately scraped off and recovered from silica with ether. The solvent was evaporated under a stream of nitrogen, and the residue was dissolved in hexane (1 mg/100 µL). Each fraction was analyzed by capillary GC under the conditions described above.

### 3.3. Hydrogenation

FAME were hydrogenated by procedures described elsewhere [35]. Briefly, 5 mg of FAME from the *S. pacifica* lipids were dissolved in 2 mL ether and 1 mg platinum oxide (Adams catalyst, Wako Pure Chemicals, Osaka, Japan) was added. The mixture was purged with hydrogen at just above atmospheric pressure, allowed to react for 1 h, and the saturated FAME were separated from the catalyst and analyzed by capillary GC, as described above.

### 3.4. Preparation of Picolinyl Ester Derivatives 

Picolinyl esters derived from the fractionated FAME were prepared according to the method of Destaillats and Angers [36]. Briefly, FAME (5 mg/mL in dry dichloromethane) were reacted with a freshly prepared mixture of 1 M potassium *tert*-butoxide in tetrahy-drofuran (100 µL) and 3-pyridylcabinol (200 µL). The reaction mixture was maintained at 40 °C for 30 min in a closed vial. After cooling to approximately 22 °C, distilled water (2 mL) and hexane (4 mL) were added. The organic phase was collected, dried over anhydrous sodium sulfate, and the effluent was evaporated to dryness under nitrogen. The residue was dissolved in 100 µL hexane for GC-electron impact ionization-MS (GC-EI/MS) analysis.

### 3.5. Preparation of 4,4-Dimethyloxazoline Derivatives

The 4,4-Dimethyloxazoline (DMOX) derivatives from the fractionated FAME were prepared according to the method of Fay and Richli [37] as follows: a total of 500 µL of 2-amino-2-methyl-1-propanol (AMP) was added to 10 mg of FAME and the mixture was incubated overnight at 180 °C under a nitrogen atmosphere. Next, the reaction tube was cooled to room temperature, and the DMOX derivatives were extracted with 5 mL of a mixture of hexane-ether (1:1, *v*/*v*). The extract was washed with 5 mL of water saturated with sodium chloride, dried over anhydrous sodium sulfate, and subsequently evaporated under nitrogen. The residue was dissolved in 100 µL hexane for GC-EI/MS analysis. 

### 3.6. H-NMR

^1^H-NMR spectra of the saturated FAME fraction containing 17% 11,12-methylene-hexadecanoic acid were acquired with CDCl_3_ as the solvent on a JEOL ECP-400 spectrometer operating at 400 MHz for ^1^H. All chemical shifts are reported relative to the chloroform peak (7.29 ppm for ^1^H-NMR).

### 3.7. FT-IR

IR spectra of monosaturated FAME fractions were recorded on thin films cast onto KBr plates using a JASCO FT/IR-4200 instrument (JASCO Corporation, Tokyo, Japan). The spectra were recorded over the wavenumber range of 4000 to 400 cm^−1^. 

### 3.8. GC-MS

Fatty acid picolinyl ester derivatives were analyzed by GC-EI/MS (Agilent Technologies 7890A GC system; Santa Clara, CA, USA) linked to a JEOL JMS-T 100GCv mass spectrometer under an ionization voltage of 70 eV at 250 °C, and using a fused silica HP-5 capillary column (30 m × 0.32 mm i.d., 0.25 µm film thickness). In the splitless injector, the interface temperatures were maintained at 230 °C, and helium was used as the carrier gas under a constant flow of 1 mL/min. The column temperature for picolinyl esters was programmed from 100 to 200 °C at 30 °C/min, then increased to 250 °C at 2 °C/min. 

DMOX derivatives were also analyzed by GC-EI/MS (Hewlett-Packard model 6890 GC system) linked to a JEOL JMS-700Tz mass spectrometer under an ionization voltage of 70 eV at 230 °C using a fused silica DB-23 capillary column (30 m × 0.25 mm i.d., 0.25 µm film thickness). The injector, in split mode (50:1), and the interface temperatures were maintained at 230 °C, and helium was used as the carrier gas at a flow rate of 1 mL/min. The temperature programming mode was as follows: 40 °C isothermal for 1 min, increasing to 160 °C at 40 °C/min, and then to 230 °C at 3.5 °C /min.

### 3.9. Mass Spectrum Data of Picolinyl Ester Derivatives

#### 3.9.1. *cis*-11,12-Methylene-Hexadecanoic Acid Picolinyl Ester 

MS *m/z* (rel. int.): 359 [M^+^] (5.2), 358 (6.3), 330 (4.8), 316 (30.2), 302 (4.9), 288 (4.7), 275 (7.1), 262 (2.5), 246 (3.0), 232 (5.0), 220 (5.1), 206 (5.3), 178 (5.1), 164 (37.5), 151 (15.1), 133 (4.8), 108 (50.0), 92 (100), 81 (4.8), 69 (18.3), 55 (49.7), 41 (35.5).

#### 3.9.2. 11-Cyclopenylundecanoic Acid Picolinyl Ester 

MS *m/z* (rel. int.): 345 [M^+^] (4.8), 344 (9.8), 304 (13.2), 276 (24.9), 262 (15.3), 248 (9.8), 234 (12.3), 220 (11.8), 206 (14.6), 193 (2.1), 178 (9.6), 164 (75.1), 151 (44.7), 120 (0.7), 108 (95.1), 92 (100), 81 (4.8), 69 (27.7), 55 (40.2), 41 (39.7).

#### 3.9.3. 13-Cyclopenyltridecanoic Acid Picolinyl Ester 

MS *m/z* (rel. int.): 373 [M^+^] (2.5), 372 (4.8), 332 (4.9), 304 (22.0), 290 (13.9), 276 (14.7), 262 (15.8), 248 (14.9), 234 (12.8), 220 (12.1), 206 (14.6), 193 (4.7), 178 (10.0), 164 (74.2), 151 (50.7), 108 (100), 92 (97.5), 83 (12.1), 69 (29.8), 55 (40.2), 41 (32.2).

#### 3.9.4. *cis-*11-Hexadecenoic Acid Picolinyl Ester 

MS *m/z* (rel. int.): 345 [M^+^] (55.3), 316 (27.2), 302 (30.4), 288 (7.3), 274 (2.9), 262 (2.2), 248 (7.5), 234 (9.5), 220 (9.0), 206 (13.8), 192 (6.5), 178 (9.1), 164 (53.5), 151 (38.6), 108 (67.8), 92 (95.1), 83 (5.6), 69 (9.0), 55 (28.2), 41 (9.5).

### 3.10. Mass Spectrum Data of DMOX Derivatives

#### 3.10.1. *cis*-11,12-Methylene-Hexadecanoic Acid DMOX 

MS *m/z* (rel. int.): 321 [M^+^] (24.2), 306 (32.1), 292 (18.3), 278 (56.0), 264 (23.7), 250 (15.7), 236 (10.1), 224 (10.4), 210 (16.0), 196 (16.3), 182 (15.9), 168 (19.2), 154 (7.8), 140 (13.2), 126 (87.8), 113 (100), 98 (9.0), 82 (7.8), 69 (11.9), 55 (32.0), 41 (8.0).

#### 3.10.2. 11-Cyclopenylundecanoic Acid DMOX 

MS *m/z* (rel. int.): 307 [M^+^] (3.8), 292 (19.6), 278 (3.9), 264 (7.6), 238 (4.0), 224 (3.8), 210 (3.6), 196 (3.8), 182 (4.8), 168 (10.1), 154 (3.5), 140 (4.7), 126 (32.4), 113 (100), 98 (6.0), 82 (3.9), 69 (11.6), 55 (12.3), 41 (3.8).

#### 3.10.3. 13-Cyclopenyltridecanoic Acid DMOX 

MS *m/z* (rel. int.): 335 [M^+^] (4.1), 320 (16.3), 306 (4.0), 292 (7.7), 266 (3.8), 252 (3.7), 238 (3.8), 224 (3.2), 210 (3.9), 196 (4.0), 182 (5.9), 168 (8.3), 154 (3.7), 140 (6.2), 126 (35.8), 113 (100), 98 (5.8), 82 (4.3), 69 (11.8), 55 (15.7), 41 (3.7).

### 3.11. NMR Spectrum Data of Saturated FAME Fraction

The following common, characteristic chemical shifts were observed in the ^1^H-NMR spectrum of the saturated FAME fraction (Figure 4). ^1^H-NMR (CDCl_3_, 400 MHz): δ 3.70 (3H, s) for -OCH_3_, 2.33 (2H, t) for -CH_2_-COO-, three multiplet signals at δ 1.77, 1.62, and 1.51 ppm for nonequivalent protons in the cyclopentane ring, 1.25–1.45 (br) for CH_2_ α to terminal CH_3_ and CH_2_ at C_4_, 1.65 (2H) for -CH_2_-CH_2_-COO-, 1.17 (2H, br) and 1.40 for CH_2_ α to the cyclopropane ring, 0.92 (3H, t) for terminal CH_3_, 0.67 (2H, br) for CH carbons and protons (C_11_, C_12_), 0.6 (1H, *trans*, ddd) and -0.3 (1H, *cis*, q) for CH_2_ of cyclopropane ring.

## 4. Conclusions

In the present study, we evaluated the fatty acid composition of the red alga *S. pacifica* (Yamada) Yoshida. Using GC-MS and NMR, we found high percentages of two unusual cyclopentyl fatty acids (11-cyclopentylundecanoic acid and 13-cyclopentyltridecanoic acid) and 16:1n-5 acid in *S. pacifica*. In particular, a cyclopropane fatty acid *cis*-11,12-methylene-hexadecanoic acid, which has not previously been found in other algae, was also found in *S. pacifica* at a significant amount (7.9% of the total fatty acids). The occurrence of *cis*-11,12-methylene-hexadecanoic acid in the alga is likely to be associated with the significant amount of *cis*-11-hexadecenoic acid. The fatty acid composition of *S. pacifica* showed quite distinctive characteristic compared to other marine algae, owing the presence of three cyclic fatty acids and 16:1n-5 acid.

## Figures and Tables

**Table 1:** Fatty acid composition of total lipids from *Solieria pacifica*.

Fatty Acid	ECL ^a^	wt %
12:0	12.00	0.05 ± 0.0
14:0	14.00	1.18 ± 0.2
Iso 15:0	14.60	0.27 ± 0.0
15:0	15.00	0.29 ± 0.0
Iso 16:0	15.37	0.17 ± 0.0
Anteiso 16:0	15.67	0.36 ± 0.0
16:0	16.00	28.36 ± 1.4
Iso 17:0	16.53	0.19 ± 0.0
17:0	17.00	0.54 ± 0.1
cy17:0 ^b^	17.39	7.89 ± 0.8
cy16:0 ^c^	17.44	7.04 ± 0.7
18:0	18.00	0.56 ± 0.1
cy18:0 ^d^	19.45	4.85 ± 0.5
ΣSaturates		51.75 ± 1.6
14:1n-5	14.27	0.24 ± 0.0
16:1n-9	16.20	0.23 ± 0.0
16:1n-7	16.26	1.02 ± 0.1
16:1n-5	16.40	8.96 ± 0.7
18:1n-9	18.28	0.80 ± 0.1
18:1n-7	18.35	0.39 ± 0.0
18:1n-5	18.49	1.64 ± 0.3
20:1n-9	20.26	0.11 ± 0.0
20:1n-7	20.32	0.20 ± 0.0
ΣMonoenes		13.59 ± 0.9
16:2n-6	16.65	0.14 ± 0.0
16:3n-3	17.30	0.03 ± 0.0
16:4n-3	17.74	0.04 ± 0.0
18:2n-6	18.77	0.27 ± 0.0
18:3n-6	19.02	0.20 ± 0.0
20:3n-6	20.91	0.39 ± 0.0
20:4n-6	21.12	23.69 ± 1.3
20:4n-3	21.29	0.05 ± 0.0
20:5n-3	21.68	9.14 ± 0.9
ΣPolyenes		33.95 ± 1.3
Others		0.71 ± 0.2

^a^ Equivalent chain length at 160 °C; ^b^ *cis*-11,12-Methylene-hexadecanoic acid; ^c^ 11-Cyclopentylundecanoic acid; ^d^ 13-Cyclopentyltridecanoic acid. ΣSaturates: sum of saturated fatty acids, ΣMonoenes: sum of monoenoic fatty acids, ΣPolyenes: sum of polyenoic fatty acids, Others: traced fatty acids.

## Data Availability

All the data presented in this study are available in this article and Supplementary Materials.

## Supplementary Materials

The following are available online, Figure S1: Partial GC profiles of total fatty acid methyl esters from *Solieria pacifica* on Omegawax 320. The column temperature was held at 160 °C, Figure S2: GC-MS profile of the picolinyl ester derivatives of the monoenoic FAME fraction of total fatty acids from *Solieria pacifica*, Figure S3: FT-IR spectrum of the methyl esters from the monoenoic acid fraction of total fatty acids from *Solieria pacifica*. A = 18:1 standard (97% *cis*, 3% *trans*), B = the methyl esters from monoenoic acid fraction.

## Sample Availability

Samples are not available from the authors.
