# Supplementary material for: Occurrence of Cis-11,12-Methylene-Hexadecanoic Acid in the Red Alga Solieria pacifica (Yamada) Yoshida
Source: Molecules. 2021 Apr 15;26(8):2286. doi: 10.3390/molecules26082286 (PMC8071341; doi:10.3390/molecules26082286)
Supplement: Supplementary file 1 [file molecules-26-02286-s001.zip › molecules-1178534-supplementary.pdf]

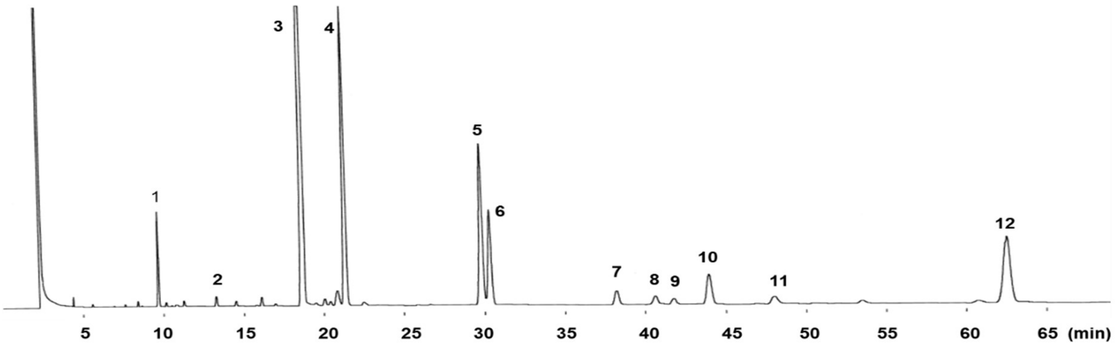

Figure S1. Partial GC profiles of total fatty acid methyl esters from *Solieria pacifica* on Omegawax 320. The column temperature was held at 160 ℃. Peak identification: **1** = 14:0; **2** = 15:0; **3** = 16:0; **4** = 16:1n-5; **5** = *cis*-11,12-methylene-hexadecanoic acid; **6** = 11-cyclopentylundecanoic acid; **7** = 18:0; **8** = 18:1n-9; **9** = 18:1n-7; **10** = 18:1n-5; **11** = 18:2n-6; **12** = 13-cyclopentyltridecanoic acid.

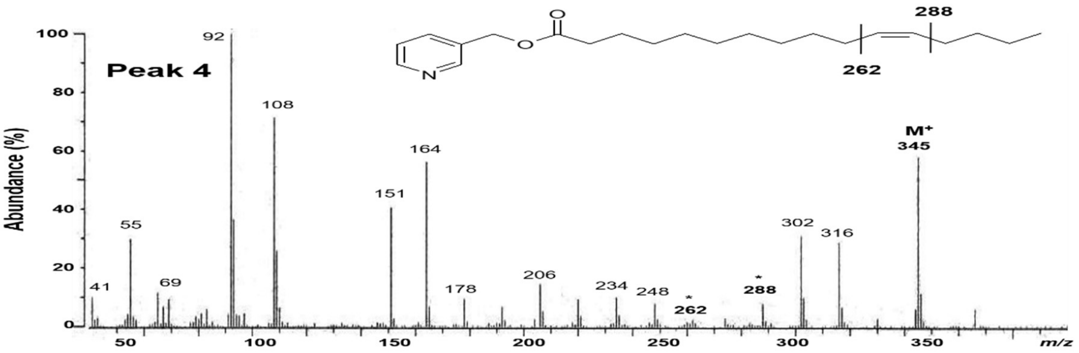

Figure S2. GC-MS profile of the picolinyl ester derivatives of the monoenoic FAME fraction of total fatty acids from *Solieria pacifica*. Peak 4 = 16:1n-5.

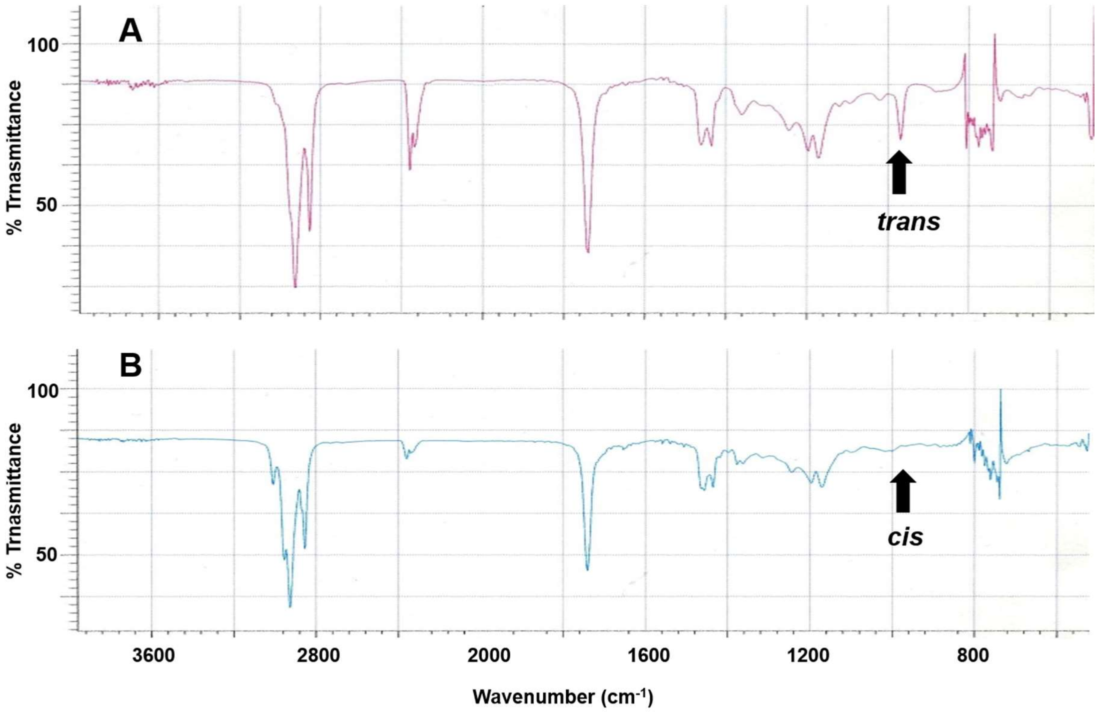

Figure S3. FT- IR spectrum of the methyl esters from the monoenoic acid fraction of total fatty acids from *Solieria pacifica*. A = 18:1 standard (97% *cis*, 3% *trans*), B = the methyl esters from monoenoic acid fraction.
